# Measurement of psychological inflexibility: an examination of the psychometric properties of the AAQ-3 compared to AAQ-II

**DOI:** 10.1186/s40359-023-01318-9

**Published:** 2023-09-30

**Authors:** Shuanghu Fang, Dongyan Ding, Mingjie Huang

**Affiliations:** School of Educational Science, Anhui Normal University, Wuhu, China

**Keywords:** Psychological inflexibility, Experiential avoidance, Discriminant validity, Measurement equivalence, College students

## Abstract

Due to the limitations of the existing measurements of experiential avoidance, we would like to check the validity of the improved version of Acceptance and Action Questionnaire–II (AAQ-II), i.e., Acceptance and Action Questionnaire–3 (AAQ-3), in Chinese content. The present study was aim to examine the construct and validity of the Chinese version of AAQ-3 in college students and provide an initial validation of this instrument to promote future cross-cultural examination of the psychological flexibility. Totally 1,572 college students were invited to complete the Chinese AAQ-3 and the related questionnaires at the same time. After one month, 380 participates were assessed with same questionnaires to examine the test-retest reliability. The results indicated a similar one-factor solution in the Chinese AAQ-3 to the original version by exploratory factor analysis, parallel analysis and confirmatory factor analysis. Internal consistency and test–retest reliability were good. According to the testing of the measurement invariance, the one-factor model was acceptable across gender (Man = 875, Girl = 697). Additionally, Chinese AAQ-3 was significantly negatively correlated with positive mental health (life satisfaction, mindful attention awareness), significantly positively correlated with negative emotions (depression, anxiety, stress), and significantly positively correlated with AAQ–II and Brief Experiential Avoidance Questionnaire (BEAQ). Besides, Chinese AAQ-3 was the strongest predictor of depression, anxiety, stress and life satisfaction compared to the AAQ-II and BEAQ. However, according to the exploratory structural equation model, the Chinese AAQ-3 demonstrated excellent discriminate validity from negative emotions. Overall, the AAQ-3 modified the limitations of the existing measurements of experiential avoidance (i.e., AAQ-II and BEAQ) as it showed better convergent validity with positive mental health indicators, better discriminant validity with negative emotions, and higher incremental validity. Therefore, the Chinese AAQ-3 is a valid measurement tool for assessing the level of experiential avoidance or psychological flexibility in Chinese college students.

## Introduction

For the past few years, Acceptance and Commitment Therapy (ACT) has received growing interest from scholars around the world for its effectiveness in improving physical and psychological issues relative to other established conventional therapies [1–4]. As a core component of the ACT, psychological flexibility, is also gaining more and more attention. Psychological flexibility can be divided into six core processes: acceptance, cognition defusion, engagement with the present moment, self as context, values, and committed action [5–7]. Psychological flexibility can assist individuals consciously accept negative life events and adversities with an open mindset, and also help people persist and act on their value-consistent goals [5, 8].

Psychological flexibility has demonstrated empirical associations with psychopathology and health [9]. Evidence-based studies have shown that flexibility is associated with various psychopathological conditions and functional outcomes [10–12]. Higher psychological flexibility is associated with improved quality of life, values and meaning seeking, positive relationships, and physical health [9, 13]. On the other hand, the opposite of psychological flexibility, i.e., psychological inflexibility, which is defined as the “rigid dominance of psychological reactions over chosen values and contingencies in guiding action” [14], is particularly salient in populations with depression and anxiety disorders [15–17]. This rigidity can manifest in various ways, reflecting six key dimensions of psychological inflexibility: cognitive fusion, experiential avoidance, lack of present moment awareness, attachment to conceptualized self, difficulty taking perspective, and lack of values clarification [5, 18]. Existing meta-analyses also suggest that psychological flexibility is positively related to individuals’ mental health and adaptive behaviors and negatively related to individuals’ negative emotional affect and problematic behaviors [19, 20]. A meta-analytic structural equation modeling examining the mechanisms of ACT found that psychological flexibility mediates changes in individual psychological symptoms [21]. These seemingly strong associations are predicated on the assumption that the measurement of psychological flexibility or psychological inflexibility is valid [9].

Owing to the critical role of psychological flexibility in the psychological and behavioral health of individuals, the construct of psychological flexibility has gained increasing attention from the outset by ACT research and other studies focusing on psychological flexibility [22, 23]. Therefore, there is a growing number of instruments that measure psychological flexibility, but one of the best known and most widely used is the Acceptance and Action Questionnaire–II (AAQ-II) [24, 25]. This scale was developed by Bond et al. to measure the level of individual psychological inflexibility (as opposed to flexibility) and experiential avoidance. Experiential avoidance refers to the process of attempting to modify the form, frequency, or situational reactivity of aversive internal experiences, such as thoughts, emotions, and physiological sensations, despite resulting in behavioral harm that is incongruent with one’s personal values and objectives, and it is one of the core constructs of psychological inflexibility and can be used as an example of psychological inflexibility [14, 18]. AAQ-II is also the most used one in China [8, 26]. Recently, an item response theory (IRT) analysis, examining the items functioning of AAQ-II within the measure and across different groups, argued that changes in scale scores may be equivalent across samples, and estimates of effect sizes can be reliably compared across different samples [24]. Also, a psychometric comparison of different psychological inflexibility measures (i.e., the Brief Experiential Avoidance Questionnaire (BEAQ), and the Comprehensive assessment of Acceptance and Commitment Therapy processes (CompACT), concluded that the other scales were not significantly better than the AAQ-II [27]. In addition, because it was the most commonly used one to assess psychological inflexibility all over the world [25, 28], AAQ-II allows for comparative studies between different cultures, races, and ethnicities. Furthermore, it only has seven items, and the short scale could reduce response time and the burden on participants to respond. These may be the reasons for its popularity among researchers.

However, AAQ-II has some limitations that cannot be ignored. Although methods based on classical test theory (CTT) have shown satisfactory psychometric properties, the AAQ-II appears to perform poorly in IRT analyses [24]. Some research suggested that the AAQ-II is subject to conceptual issues and does not have sufficient discriminant validity [27, 29]. In addition, the AAQ-II focuses and assesses more on distress and negative emotions [29, 30]. IRT analyses argued that the weak discriminant validity may be due to the unclear wording, and revealed that generally worded items did not perform well than items that specify the function of an internal experience [24]. For example, items 3 (i.e., “I worry about not being able to control my worries and feelings”) and 4 (i.e., “My painful memories prevent me from having a fulfilling life”) in AAQ-II which inquired more concretely about the function of life events, appeared to show greater discrimination. Conversely, more broadly worded items, like items 2 (i.e., “I’m afraid of my feelings”), 6 (i.e., “It seems like most people are living their lives better than I am”) or 7 (i.e., “Worries get in the way of my success”), may provide less information and reflect more general population-specific responses.

If the above problem of AAQ-II can be solved, it would be able to provide useful information of psychological inflexibility and experiential avoidance. So, there is AAQ-3, which is an improvement of AAQ-II and adjusted some problems of wording of AAQ-II [27]. Like the AAQ-II, the AAQ-3 also has seven items. But the wording of all items in AAQ-II has been modified to improve clarity and item-level functioning. Each item of AAQ-3 is rated on a seven-point scale ranging from 1 (never true) to 7 (always true), and higher scores indicate more psychological inflexibility like AAQ-II. Compared with AAQ-II and BEAQ, AAQ-3 had stronger discriminant validity [27]. Thus, the AAQ-3 could be more suitable to detect psychological inflexibility. However, the vast majority of ACT research or psychological flexibility studies have been conducted in Western countries and in English.

Nowadays, there is a growing attention among Chinese researchers that are focusing on the effects of psychological flexibility on individuals [31, 32]. In fact, some of the core principles of ACT can be traced back to Chinese culture, such as the Tao Te Ching, a book written by Lao Tzu 2,500 years ago, which encourage individuals to accept things as the way they are [33, 34]. In addition, due to the influence of traditional culture, the Chinese do not advocate avoidance or escape from adversity, and tolerance and acceptance is the national character of Chinese people [35]. Existing Chinese idioms and colloquialisms often reflect this characteristic, for example, “Take things as they come”(随遇而安), “You can run but you can never hide”(躲得了初一, 躲不了十五). Therefore, there may be some cultural differences need to be explored in the level of experiential avoidance between the Chinese and people from countries without this context.

Based on the possible cultural differences described above, introducing the AAQ-3 to China will not only enabled researchers to examine the measurement indicators and applicability of the questionnaire, but also provide the necessary tool and perspectives for cross-cultural research. It could also provide a more valid measuring tool for ACT research in China. The primary purpose of this paper is to provide Chinese researchers or research in China with a tool having clearer, more specific items, and sufficient discriminant validity to measure experiential avoidance, i.e., to translate, validate, and test the gender invariance of the Chinese version of the AAQ-3.

## Methods

### Participants

In this study, a convenience sampling method was used and the data were obtained through an Internet applet (sojump-Wenjuanxing). The first author of this paper contacted the student administration of six universities, explained to them in detail the purpose of the study and the research procedures, and obtained their consent. The first author of this study conducted online training for the counselors involved in the study and then forwarded the questionnaire link to the counselors, who explained the study procedures in detail to the students. Written consent was obtained from each participant before completing the questionnaire (online document collection applet).

The survey was conducted between November 2021 and January 2022. A total of 1,640 questionnaires were collected. In order to prevent the participants from answering regularly, “specified option questions” were set. After screening invalid questionnaires, there were 1,572 valid questionnaires, with an effective response rate of 95.85%. Among them, 875 were male and 697 were female; aged 20.15 ± 1.21 years in average; 739 were majoring in science and technology, 688 in literature and history, and 145 in other majors; 913 were in rural areas and 659 in urban areas. One month later, 390 people were invited again for retesting, and 380 valid questionnaires were received.

### Procedure

The first author of this study obtained the consent of the original author to translate and revise the AAQ-3. The translation process involves the translation of English into Chinese (forward translation) [36] and Chinese into English (back translation) [37]. First, the first author of this study, who is fluent in both Chinese and English, translated the AAQ-3 into Chinese and modified ambiguous terms in order to obtain a preliminary draft of the Chinese version. Second, two more English-speaking psychologists translated the Chinese version draft back into English. We acquired the back-translated English version of the scale by considering the comments of two specialists. Following that, through multiple online meetings, the three experts who participated in the translation and back-translation formed an expert group to discuss and compare the differences between the original English version, the English version after back-translation, and the first draft of the Chinese version. They revised and updated the initial draft of the Chinese version and received the scale’s second draft in Chinese. In addition, we asked thirty undergraduates and postgraduates from a university library or a self-study room at random to evaluate and offer feedback on the readability and comprehensibility of each item in the Chinese version of the second draft. After carefully evaluating all relevant evaluations and suggestions, the first author of this study updated and examined the Chinese version of AAQ-3 once more, resulting in the final Chinese version of AAQ-3.

### Measures

#### Acceptance and Action Questionnaire-3

This scale is a modified version of the AAQ-II and can be used to measure psychological inflexibility in individuals [27]. There are 7 items (e.g., “How I react to emotions causes problems in important areas of my life”), scored on a 7-point Likert scale ranging from 1 (never true) to 7 (always true). Total scores are summed and higher scores indicate higher levels of experiential avoidance in individuals.

#### Acceptance and Action Questionnaire-II

The scale was developed to measure the level of individual experiential avoidance [14]. A revised Chinese version with 7 items was used in this study [26]. Items (e.g., “Emotions cause problems in my life”) are rated on a 7-point scale ranging from 1 (never) to 7 (always). The scores were averaged and higher scores indicated higher levels of experiential avoidance. In the present study, the scale has good internal consistencies, with Cronbach’ s alpha value of 0.85 [0.82, 0.87]. See Appendix 1 for a comparison of AAQ-3 and AAQ2 in English and Chinese.

#### Satisfaction with Life Questionnaire (SWLS)

The scale was developed to measure life satisfaction and subjective well-being [38]. A revised Chinese version was used in this study [39], which was widely adopted in China to measure individual satisfaction with life [40]. The scale consists of 5 items (e.g., “If I could live my life over, I would change almost nothing”), and participants rated each item on a 7-point scale ranging from 1 to 7 (strongly disagree to strongly agree). Responses were averaged so that higher scores corresponded to higher life satisfaction. The scale has good psychometric reliability, with a Cronbach’s alpha of 0.78 in its original study. In the present study, it showed a Cronbach alpha of 0.89 [0.87, 0.91].

#### Mindful attention awareness scale (MAAS)

The scale can be used to measure the degree and level to which a person can be aware of thoughts, motivations, emotions, and sensory and perceptual stimuli in daily life [41, 42]. It consists of 15 items (e.g., “I find myself doing things without paying attention”). Participants rated each item on a 6-point scale ranging from 1 to 6 (almost always to almost never). Responses were averaged so that higher scores corresponded to higher level of mindfulness. The scale showed an internal consistency of 0.85 [0.83, 0.87] at present study.

#### Brief Experiential Avoidance Questionnaire (BEAQ)

The BEAQ is a 15-item measure of experiential avoidance [43]. The revised Chinese BEAQ has two dimensions, i.e., cognitive avoidance and behavioral avoidance with same items [44]. Participates response each item using a 6-point Likert scale from 1 (strongly disagree) to 6 (strongly agree). Items include “Quick to leave situations that make me uneasy” and “Try to put unpleasant memories out of mind”. The scores were averaged and higher scores indicated higher levels of experiential avoidance. Internal consistency for the BEAQ in the current study was 0.81 [0.79, 0.84].

#### Depression anxiety stress scales (DASS-21)

The DASS-21 was designed to measure individual psychological distress, i.e., depression, anxiety and stress [45]. The revised Chinese version was applied in this study, with twenty-one items and three dimensions [46]. A 4-point scale ranging from 0 (“did not apply to me”) to 3 (“applied to me very much”) is presented to each item, with higher scores indicating severe psychological distress. Cronbach’s alpha for the total scale and its subscales, i.e., depression, anxiety, and stress were 0.94 [0.93, 0.95], 0.88 [0.86, 0.89], 0.83 [0.93, 0.95] and 0.86 [0.86, 0.88] respectively.

### Data analysis

Statistical analysis was conducted using SPSS 22.0 and Mplus7.4. First, the entire sample(*n* = 1,572) was randomly divided into 2 subsamples using the SPSS random number generator to generate random numbers in the range from 0 to 1000 starting with a fixed value of 20,220,203. Subsequently, item analysis and exploratory factor analysis (EFA) were performed on sample one (*N* = 786). Unweighted leasts quares method and Varimax orthogonal rotation was used to identify the factor structure of the AAQ-3. Bartlett’s test of sphericity and the Kaiser–Meyer–Olkin (KMO) statistic were used to assess the appropriateness to perform factor analysis on the items. The data were considered suitable for factor analysis if Bartlett’s test was significant and the KMO statistic was ≥ 0.8 [47].

And confirmatory factor analysis was performed on sample two (*N* = 786). Gender equivalence was then tested for the total sample (*N* = 1,572). Predictive validity and retest reliability were tested using a retest sample (*N* = 380). In addition, Pearson correlation was used to test concurrent validity and convergent validity (*N* = 1572). Exploratory structural equation modeling (ESEM) was used to test discriminant validity (*N* = 1572), and incremental validity was tested by hierarchical regression (*N* = 1572).

The following parameters were used to identify the model fit: *χ2, CFI* (Comparative Fit Index), *TLI* (Tucker-Lewis index), *RMSEA* (Root Mean Square Error of Approximation), and *SRMR* (Standardized Root Mean Square Residual). The.

values of *CFI* and *TLI* > 0.90 were judged to a good fit and the values > 0.80 were judged to an acceptable fit [48]. The values of *RMSEA* and *SRMR* < 0.08 were judged to an acceptable fit and *SRMR* < 0.06 were judged to an excellent fit [49]. The data were consistent with the normal distribution (mean ± standard deviation), and the *t* test was used for comparison between groups.

## Results

### Item analysis

The total scores of AAQ-3 in sample one (N = 786) were ranked. Individuals with score at 27th percentile or 73th percentile of the distribution of scores were assigned to low or high subgroups respectively. The results of the independent samples *t*-test showed that the high and low groups differed significantly in each item (*ρ* < 0.001), indicating that each item had good discriminant validity. Second, Pearson correlation analysis was used to obtain the correlation coefficients of each item with the total scores of AAQ-3. The correlation analysis showed that each item had a high consistency with the scale. See Table 1.

**Table 1:** Descriptive statistics, item analysis and factor loading (N = 1572)

Items	Descriptive statistics				Differences between groups (M ± SD)			I-T	Factor loading
	*M*	*SD*	Skewness	kurtosis	High	Low	*t*		AAQ-3
Item 1	3.40	1.41	0.17	-0.24	4.46 ± 1.01	2.15 ± 0.99	25.19^***^	0.67	0.71
Item 2	3.78	1.36	-0.03	-0.07	4.74 ± 1.03	2.46 ± 1.05	25.24^***^	0.74	0.78
Item 3	3.67	1.49	0.13	-0.35	4.74 ± 1.58	2.27 ± 1.09	25.17^***^	0.70	0.75
Item 4	3.01	1.42	0.44	-0.14	4.19 ± 1.16	1.66 ± 0.77	30.33^***^	0.74	0.79
Item 5	3.50	1.45	0.15	-0.46	4.63 ± 1.10	2.32 ± 0.93	29.12^***^	0.72	0.76
Item 6	3.54	1.34	0.17	-0.18	4.51 ± 1.04	2.32 ± 1.00	24.69^***^	0.68	0.72
Item 7	3.23	1.38	0.14	-0.11	4.60 ± 1.01	2.16 ± 0.93	28.68^***^	0.73	0.77

Note: ^*^, *p* < 0.05;^**^, *p* < 0.01; ^***^, *p* < 0.001, I-T = The correlations between items and total scores of AAQ-3

### Exploratory factor analysis

**Fig. 1:**
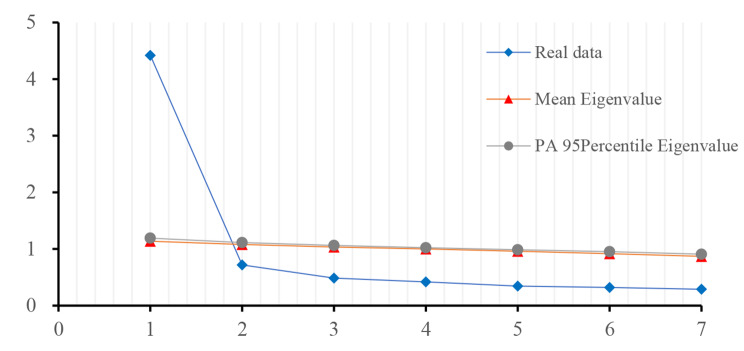
Scree plot for parallel analysis

Exploratory factor analysis was performed on sample one (n = 786). First, *KMO* sample fit test and Bartlett’s sphericity test were performed. The results, *KMO* = 0.90, *χ*^*2*^ = 3031.06, *df* = 21, *ρ* < 0.001, indicated that the data were suitable for exploratory factor analysis. Unweighted leasts quares method and Varimax orthogonal rotation were used to perform factor analysis on the item. The Cattell’s scree test with a parallel analysis recommends to retain only those factors whose eigenvalues are greater than that from the random data [50]. Then, parallel Analysis was then used to further determine the number of factors accurately by comparing the mean or 95th percentile of the eigenvalues from real data with those from the random data [51, 52]. The results of the parallel analysis showed that the eigenvalues of the real data on the 2nd factor (0.72) were smaller than the mean (1.08) or 95th percentile (1.11) of the eigenvalues from the random data. The eigenvalue for the one factor was 4.42, and the factor loadings of each item ranged from 0.71 to 0.79.so it was reasonable to retain 1 factor. The factor structure and content were consistent with the original scale (see Table 1; Fig. 1).

### Reliability analysis

The proportions of students who obtained the lowest score and the highest score were extremely low for AAQ-3 at both time points (< 2.0%). This result suggests the absence of floor and ceiling effects. The Cronbach alpha coefficient of the Chinese AAQ-3 was 0.90 [0.88,0.91], McDonald’s omega (*ω*) = 0.88 [0.90, 0.92]. And the Guttman’s split-half reliability, Guttman’s *λ2* and *λ6* were 0.79, 0.90 and 0.90 respectively. And the retest reliability (*ICC*) was 0.65, 95%*CI* (0.60,0.71). These indicated that the Chinese AAQ-3 has good internal consistency and retest reliability.

### Confirmatory factor analysis

We conducted a confirmatory factor analysis on sample two (*N* = 786), using all items as indicators of the only latent variable. The results showed a good model fit indices of the one-factor model (*χ*^*2*^ = 82.61, *df* = 14, *CFI* = 0.98, *TLI* = 0.97, *RMSEA* = 0.08, *SRMR* = 0.02), which indicated the reasonableness of the one-factor construct of the Chinese version of AAQ-3.

### Measurement equivalence

Measurement equivalence was tested on the complete sample (*N* = 1,572). The Chinese version of AAQ-3 was tested for cross-gender equivalence by constructing a multi-group model. In the configural invariance model, the model fit was acceptable and met the conditions of the next equivalence analysis. We also set the factor load equivalence (metric invariance model), the index intercept equivalence (scalar invariance model), and the error variances equivalence (strict invariance model) based on the previous model. It was found that the *ΔCFI* and Δ*RMSEA* were less than 0.01 between the configural equivalence and between the weak measurement equivalence, and between the comparison of weak measurement equivalence with the strong measurement equivalence, and between the comparison of strong invariance with the strict invariance [53, 54]. Therefore, the results indicated that the items of Chinese version of AAQ-3 operate nearly identically across male and female, supporting the direct comparison of scores across those two primary genders. For details, see Table 2.

**Table 2:** Measurement equivalence cross-gender (N = 1572)

	*SBc* ^*2*^	*df*	*CFI*	*TLI*	*SRMR*	*RMSEA[90%CI]*	*ΔCFI*	*ΔRMSEA*
Male (*N* = 875)	45.65	11	0.989	0.980	0.019	0.060(0.043,0.079)		
Female (*N* = 697)	41.35	11	0.989	0.978	0.018	0.063(0.043,0.084)		
Configural model	87.00	22	0.989	0.979	0.019	0.061(0.048,0.075)		
Metric invariance	97.81	28	0.988	0.982	0.029	0.056(0.044,0.069)	0.001	0.005
Scalar invariance	103.94	34	0.988	0.985	0.029	0.051(0.040,0.063)	0.001	0.001
Strict invariance	119.97	41	0.987	0.986	0.035	0.050(0.039,0.060)	0.001	0.001

### Convergent validity

Pearson correlation analysis was used to calculate the convergent validity. The results showed that the total scores of AAQ-3 in T1 were significantly positively correlated with the scores of depression, anxiety, stress, AAQ-II and BEAQ in T1, and negatively correlated with the scores of MAAS and SWLS in T2; the total scores of AAQ-3 in T2 were significantly correlated with the scores of depression, anxiety, stress and SWLS in the same direction as T1. This indicated that the Chinese version of the AAQ-3 has good convergent validity. For details, see Table 3.

**Table 3:** Convergence validity of the Chinese AAQ-3

	AAQ- II	BEAQ	MAAS	SWLS	DASS-depression	DASS-anxiety	DASS-stress
TI (*N* = 1572)							
AAQ-3	0.40^***^	0.37^***^	-0.43^***^	-0.35^***^	0.50^***^	0.51^***^	0.54^***^
T2(*N* = 380)							
AAQ-3	0.35^***^	0.23^***^	-0.23^***^	-0.16^***^	0.31^***^	0.25^***^	0.33^***^

Note: T1 = Time 1; T2 = Time 2; *p < 0.05;**p < 0.01;***p < 0.001

### Discriminant validity

To assess the discriminant validity of the Chinese version of AAQ-3 with negative emotions (depression, anxiety, and stress), the exploratory structural equation model (ESEM) was used to further validate the fit of the data for the full sample (*N* = 1,572) [55]. The model fit was good (*χ*^*2*^ = 1685.42, *df* = 272, *CFI* = 0.94, *TLI* = 0.92, *RMSEA* = 0.06, *SRMR* = 0.03). The level of factor loadings for each AAQ-3 item was high (0.52–0.82), and the largest cross-loadings came from the fourth item in DASS-Anxiety (0.15), but this value was much lower than that on DASS-Anxiety (0.51). Therefore, this indicated that the Chinese version of AAQ-3 has good discriminant validity with negative emotions (DASS-Depression, DASS-Anxiety, DASS-Stress), as shown in Table 4.

**Table 4:** The discrimination validity and standardized factor loadings of exploratory structural equation model (N = 1572)

Items	EA	DE	AN	ST
EA1	**0.79**	0.05	-0.01	-0.07
EA2	**0.80**	-0.05	-0.07	0.08
EA3	**0.73**	-0.05	0.01	0.08
EA4	**0.82**	0.14	0.03	-0.15
EA5	**0.75**	0.01	0.04	0.01
EA6	**0.52**	0.02	-0.04	0.11
EA7	**0.72**	-0.02	0.06	0.04
DE1	-0.04	**0.45**	0.03	0.09
DE2	0.03	**0.53**	0.13	0.19
DE3	0.09	**0.47**	0.05	0.22
DE4	0.03	**0.48**	0.20	0.12
DE5	-0.02	**0.66**	-0.01	0.09
DE6	-0.01	**0.79**	0.03	-0.06
DE7	0.02	**0.87**	-0.11	-0.04
AN1	-0.01	0.13	**0.48**	0.27
AN2	-0.02	0.03	**0.80**	-0.09
AN3	-0.01	0.05	**0.63**	0.01
AN4	0.15	0.02	**0.51**	0.20
AN5	0.01	0.10	**0.50**	0.04
AN6	0.05	0.06	**0.54**	0.10
AN7	0.06	0.04	**0.53**	0.18
ST1	0.01	-0.05	-0.10	**0.73**
ST2	0.01	0.06	-0.05	**0.47**
ST3	0.01	0.01	0.08	**0.56**
ST4	-0.01	0.14	0.12	**0.65**
ST5	0.03	0.13	0.05	**0.57**
ST6	0.04	0.12	0.02	**0.56**
ST7	0.04	0.06	0.03	**0.60**

Note: EA is the AAQ-3 scores; DE is the DASS-Depression scores; AN is the DASS-Anxiety scores; ST is the DASS-Stress scores. The bolded part is the highest factor loading.

### Incremental validity

This study used hierarchical regression analysis to test the incremental validity of the Chinese version of AAQ-3. That is, to test whether the Chinese version of AAQ-3 can explain SWLS and DASS-21 beyond AAQ-II and BEAQ. The results showed that the explanatory power (*ΔR*^*2*^) of AAQ-3 on SWLS, DASS-depression, DASS-anxiety and DASS-stress remained significant after controlling for the effects of AAQ-II and BEAQ. Moreover, the standardized regression coefficients (*β*) of AAQ-II and BEAQ on the dependent variables were significantly reduced after the inclusion of AAQ-3. This indicated that the AAQ-3 was a stronger predictor of SWLS, DASS-depression, DASS-anxiety, and DASS-stress compared to the AAQ-II and BEAQ. See Table 5.

**Table 5:** Incremental validity of the Chinese version of AAQ-3 (N = 1572)

	*B*	*SE*	*β*	*T*	*R* ^*2*^	*ΔR* ^*2*^
**SWLS**						
Step1					0.07^***^	
AAQ–II	-0.26^***^	0.02	-0.26^***^	-10.54		
BEAQ	-0.03	0.02	-0.04	-1.59		
Step2					0.14^***^	0.07^***^
AAQ–II	-0.15^***^	0.03	-0.15^***^	-5.84		
BEAQ	0.04	0.02	-0.05	-2.29		
AAQ-3	-0.23^***^	0.02	-0.31^***^	-11.20		
**DASS-depression**						
Step1					0.14^***^	
AAQ–II	0.17^***^	0.02	0.27^***^	11.33		
BEAQ	0.11^***^	0.01	0.22^***^	9.34		
Step2					0.27^***^	0.13^***^
AAQ–II	0.08^***^	0.02	0.12^***^	5.00		
BEAQ	0.04	0.01	0.09^***^	3.79		
AAQ-3	0.21^***^	0.01	0.42^***^	16.74		
**DASS-anxiety**						
Step1					0.17^***^	
AAQ–II	0.20^***^	0.01	0.33^***^	14.18		
BEAQ	0.09^***^	0.01	0.21^***^	9.21		
Step2					0.29^***^	0.12^***^
AAQ–II	0.12^***^	0.01	0.19^***^	8.00		
BEAQ	0.04	0.01	0.09	3.81		
AAQ-III	0.19^***^	0.01	0.40^***^	16.16		
**DASS-stress**						
Step1					0.25^***^	
AAQ–II	0.29^***^	0.02	0.40^***^	17.95		
BEAQ	0.13^***^	0.01	0.25^***^	11.45		
Step2					0.36^***^	0.11^***^
AAQ–II	0.19^***^	0.02	0.26^***^	11.70		
BEAQ	0.07^***^	0.01	0.13^***^	6.02		
AAQ-3	0.22^***^	0.01	0.39^***^	16.37		

### Predictive validity

Linear regression analysis showed that the total score of AAQ-3 at T1 significantly negatively predicted SWLS, and significantly positively predicted depression, anxiety, and stress at T2 after controlling for the effects of gender and age. This suggested that the Chinese version of the AAQ-3 had good predictive validity. For details, see Table 6.

**Table 6:** Predictive validity of the Chinese version of AAQ-3 (N = 380)

Dependent variables (T2)	Independent variables (T1)	*B*	*SE*	*β*	*T*	*R* ^*2*^
SWLS	AAQ-3	-0.26^***^	0.02	-0.35^***^	-14.47	0.12^***^
DASS-depression	AAQ-3	0.25^***^	0.01	0.50^***^	22.63	0.25^***^
DASS-anxiety	AAQ-3	0.24^***^	0.01	0.50^***^	23.02	0.26^***^
DASS-stress	AAQ-3	0.30^***^	0.01	0.54^***^	25.10	0.29^***^

## Discussion

As ACT research and psychological flexibility studies have become popular worldwide, more and more studies have begun to focus on the psychological flexibility of Chinese people [31, 32]. Due to traditional cultural influences, Chinese people may differ from groups in other cultural context in the level of experiential avoidance [56]. However, this requires more precise measurement tools to enrich the related studies. For this purpose, we tried to introduce AAQ-3.

The AAQ-3 is an improvement on the AAQ- II, with more specific and clear items. The aim of this study was to examine the psychometric properties of the Chinese AAQ-3 among Chinese university students. The findings indicated that the Chinese AAQ-3 is consistent with the original study [27] in terms of content validity and structure validity. Specifically, it has good predictive validity for both positive and negative mental health indicators, and has high internal consistency and retest reliability. In addition, the Chinese AAQ-3 filled the limitations of the existing measurements of experiential avoidance (i.e., AAQ-II and BEAQ) as it showed better convergent validity with positive mental health indicators, better discriminant validity with negative emotions, and higher incremental validity. Therefore, the Chinese AAQ-3 is a valid measurement tool for assessing the level of experiential avoidance or psychological flexibility in Chinese college students.

As the most broadly used instrument for measuring psychological flexibility in the world, the AAQ-II has been widely validated for its reliability and validity [26, 57, 58]. For example, the Chinese version of the AAQ-II was found to have good psychometric properties in college students [26]. Benefiting from the widespread use of AAQ-II, its potential problems have also come to light, such as the problem of discriminant validity, i.e., the inability to distinguish it from negative emotions [29, 30]. This is the main criticism of AAQ-II. Thus, Ong et al. modified AAQ-II, and the modified one was AAQ-3, which clarified the items and increased the function of item level [27]. For example, some items in AAQ-II referring to valued living were further clarified, and the emotion items were modified as reflections of emotions.

This study found that AAQ-3 had better discriminant validity, convergent validity, and incremental validity, than that of AAQ-II. In addition, the current research results also show that AAQ-3 has good retest reliability and predictive validity. The results of the correlation analysis were consistent across time, which is consistent with previous studies [27, 33, 59, 60]. The present study also found that compared with BEAQ, AAQ-3 had stronger discriminant validity, which is also consistent with previous studies [27]. All of the above suggested that the changes made by AAQ-3 in response to AAQ-II were successful.

There were some limitations in the current study. In the context of the study, the AAQ-3 is primarily designed to measure psychological inflexibility, which captures the rigid and inflexible responses to internal experiences that hinder the ability to live a valued life. Although it is related to psychological flexibility, it focuses on assessing the aspects of inflexibility rather than the overall flexibility construct. Psychological inflexibility is not the same as lack of psychological flexibility, and they are two distinct constructs [9]. Therefore, when using it, one needs to be careful about the target variables they measure, and future research could consider exploring the reliability of other instruments that measure psychological flexibility and its subcomponents in China to provide a more comprehensive understanding of these constructs. The sample of this study was mainly college students, which limited the external validity of this study, although they came from different cities. Future studies should examine its validity and reliability in different groups (i.e., clinical samples and community samples). Additionally, all data were gathered through online questionnaires and self-report scales, which raised the risk of common method variance, and future research should use experiments, clinical interviews, and other multiple informants to address this limitation. Besides, future research may benefit from longitudinal follow-up measures to explore the trend of psychological flexibility, which was the limitation of this study. Finally, this study did not report differences in AAQ-3 scores between the Chinese and other countries with different cultural contexts. Some scholars suggested that collectivist cultures promote a more psychologically inflexible pattern of behavior among individuals in comparison to individualistic cultures [61], and there may be different response tendencies between two cultures that cultural factors may impact the expression and experience of psychological inflexibility [61, 62]. Thus, future studies could benefit a lot from such a comparison between the Chinese and other countries with different cultural contexts.

In conclusion, this study contributed to the adaptation of the original AAQ-3 to a Chinese version. The results suggested that it can be applied to assess the level of experiential avoidance or psychological inflexibility in Chinese university students. This study provides practitioners and researchers with a suitable measurement tool for studying the cultural difference in experiential avoidance. It also can further promote the in-depth study of ACT in China.

## Data Availability

The datasets used and/or analyzed during the current study are available from the corresponding author upon reasonable request.
